# Protective effects of *Bifidobacterium longum* subsp. *longum* BL21 against cyclophosphamide-induced reproductive dysfunction in zebrafish

**DOI:** 10.1038/s41598-025-97721-w

**Published:** 2025-04-18

**Authors:** Yao Dong, Jiazhi Zhou, Hairui Tian, Zhonghui Gai, Kang Zou, Quanwei Wei, Mei Han

**Affiliations:** 1 Germline Stem Cells and Microenvironment Lab, College of Animal Science and Technology, Nanjing Agricultural University, Nanjing, 210095 China; 2 Stem Cell Research and Translation Center, Nanjing Agricultural University, Nanjing, 210095 China; 3 Department of Food Quality and Safety, Shanghai Business School, Shanghai, 200235 China; 4 Department of Research and Development, Wecare Probiotics Co., Ltd., Suzhou, 215200 China

**Keywords:** Probiotic, Male infertility, Zebrafish (*Danio rerio*), Sperm vitality, Hormonal modulation, Immunology, Microbiology

## Abstract

This study aimed to investigate the effects of probiotic *Bifidobacterium longum* subsp. *longum* BL21 on reproductive health in zebrafish (*Danio rerio*), focusing on hormonal modulation, sperm vitality, and overall reproductive function improvement. Adult male zebrafish of the wild-type AB strain were divided into four groups: control (CTL), model control with cyclophosphamide-induced oligospermia (CS), probiotic-treated (BL21), and a positive control treated with clomiphene citrate (CC). All groups, except CTL, were exposed to cyclophosphamide from day 1 to day 9 to induce reproductive dysfunction, with subsequent monitoring until day 16. Key metrics assessed included body and testicular weight, sperm vitality, male courtship behavior, hormone levels, sperm DNA fragmentation, and gene expression of PCNA, NANOG, ZBTB16, mTOR, DDX4, CYP26A1, and ALDH1A2. In this study, BL21 demonstrated significant therapeutic potential in treating reproductive dysfunction in zebrafish. Metabolomic analysis revealed that BL21 influenced crucial pathways, notably upregulating mTOR signaling and isoflavone biosynthesis, while downregulating the TCA cycle and pyruvate metabolism, suggesting a broad biochemical impact. The probiotic treatment notably improved testicular weight (*p* < 0.01) and sperm count and vitality (*p* < 0.001) compared to the cyclophosphamide-induced model control, underscoring its efficacy in enhancing reproductive parameters. Additionally, BL21 intervention led to a marked increase in chasing behavior and sex hormone levels, surpassing those of the positive control. The treatment also significantly reduced sperm DNA fragmentation (*p* < 0.001) and increased the expression of genes crucial for spermatogenesis and testicular function (*p* < 0.05), confirming its potential to restore reproductive health at multiple biological levels. These results highlight the promise of BL21 as a multi-faceted agent for improving reproductive health, warranting further investigation in clinical settings. Probiotic BL21 enhances reproductive parameters in zebrafish by modulating hormone levels, improving sperm quality, and positively affecting reproductive behavior. These findings suggest potential therapeutic applications of probiotics in managing reproductive health issues.

## Introduction

Male infertility (MI) is a global health concern affecting approximately 8–12% of couples worldwide, with male factors implicated in about half of these cases^1^. Its underlying causes are diverse, including environmental factors, endocrine disorders, immunological dysfunctions, and genetic factors^2^. Medically, male reproductive dysfunction generally refers to functional abnormalities or suboptimal performance in one or more components of the reproductive system, encompassing a range of conditions that may affect fertility, such as abnormal sperm, poor semen quality, and related hormonal imbalances^3^. This not only causes significant harm to individuals but may also lead to other secondary diseases. Studies indicate that about 46% of male infertility patients exhibit symptoms of oligospermia, characterized by significantly reduced sperm count and motility^4^. Statistics show that sperm quality among Chinese men declines by 1% annually, while sperm density and motility rates in Western countries decrease by 2.6% and 0.3% per year, respectively^5,6^. Male reproductive health issues not only impact the quality of individual lives but also pose a significant socioeconomic burden. Additionally, male reproductive disorders have a serious impact in the field of animal husbandry, where poor semen quality, reduced libido, or abnormalities in reproductive organs directly affect the productivity and economic benefits of the livestock industry. Reproductive health problems in breeding bulls, boars, or rams not only determine breeding efficiency but also profoundly affect the supply chain of meat, dairy products, and other animal products^7,8^. These challenges increase the reliance on artificial insemination and other reproductive technologies, thereby raising production costs. Thus, research into human and animal male reproductive disorders encompasses not only medical and animal science fields but must also consider its extensive economic and social impacts^8^. Public health and animal welfare policies must adopt a multidisciplinary approach to address this challenge, aiming to improve the quality of life for humans and animals and ensure the sustainability of the livestock industry. In this context, the exploration of safe and non-toxic bioactive compounds for treating reproductive disorders becomes particularly important.

Traditional treatments for male reproductive disorders primarily include hormone therapy, such as testosterone replacement and the use of gonadotropin-releasing hormone analogs and antagonists, aimed at regulating hormone levels in patients^9^. Additionally, surgical interventions, such as varicocelectomy and vasectomy reversal, are commonly used to address physical obstructions in the reproductive tract^10,11^. With advancements in biomedical sciences, biologics targeting specific inflammatory or immunoregulatory factors have emerged as cutting-edge options for treating male reproductive disorders, favored for their targeted action and minimal side effects. Recently, probiotics, such as the *Bifidobacterium longum* subsp. *longum* BL21 (BL21), have emerged as a novel therapeutic approach^12^, demonstrating potential through the regulation of gut health, optimization of hormone production, reduction of chronic inflammation^13–15^, and improvement of the overall reproductive environment. These effects could not only directly influence systemic metabolism and hormone levels but also enhance male reproductive health by improving immune status. Thus, integrating traditional and modern therapeutic approaches with probiotic interventions may open new avenues for treating male reproductive disorders.

Traditionally, research into reproductive disorders has relied on mammalian models, such as mice. However, the zebrafish is increasingly becoming an important tool in reproductive biology research due to its unique advantages. The reproductive system of zebrafish shares high structural and functional similarities with humans^16^, including spermatogenesis, mechanisms of hormone secretion, and the microanatomy of the testes, making it an ideal model organism. Additionally, the commonality in hormonal regulation and genetic mechanisms between zebrafish and humans provides a unique advantage in understanding human reproductive disorders and developing new therapeutic approaches. In this study, we used cyclophosphamide (CP) to induce a male reproductive dysfunction model in zebrafish. Cyclophosphamide, used in chemotherapy for cancer, is known to damage the reproductive system, particularly increasing the risk of oligospermia or azoospermia and closely associated with the pathological processes of reproductive dysfunction^17^. With the zebrafish model, we can study the effects of CP on spermatogenesis in a controlled environment and evaluate the potential therapeutic effects of Bifidobacterium longum subspecies longum BL21 on these disorders.

Therefore, this study aims to utilize BL21 in an intervention on a cyclophosphamide-induced zebrafish model of reproductive dysfunction, to evaluate its potential effects on the morphology and function of reproductive organs as well as on hormone levels. The research conducted in two phases: initially, the role of BL21 in key metabolic pathways were preliminarily explored through in vitro metabolomics approaches; subsequently, the efficacy of BL21 in restoring the morphology and improving the function of reproductive organs, as well as in the regulation of sex hormones, were assessed using a male zebrafish model of reproductive dysfunction. This approach not only helps to deepen our understanding of the impact of BL21 on male reproductive health but also provides a scientific basis for potential clinical applications.

## Methods

### Instruments, consumables, and reagents

Key instruments included precision electronic balance (OHAUS, USA), fluorescence microscope (ZEISS, Germany), multifunctional microplate reader (TECAN, Austria), flow cytometer (Agilent Technologies, USA), and real-time PCR system (BIO-RAD, Singapore). Main reagents were cyclophosphamide monohydrate (Aladdin, China), sperm vitality staining kit (Shanghai Jimei Gene Pharmaceutical, China), zebrafish hormone ELISA kits (Shanghai Enzyme-linked Biotechnology, China), quantitative PCR reagents (Bio-Rad, USA), and PBS (Biosharp, China).

### Preparation and whole-target metabolomic analysis of stain BL21

The handling and cultivation of BL21 were conducted under aseptic conditions. The strain was provided by Wecare Probiotics Co. Ltd. For the cultivation of the BL21 strain, a modified MRS medium was used, supplemented with 0.1% L-cysteine hydrochloride to optimize growth conditions^18^. After incubation at 37 °C in an anaerobic environment for 22–24 h, the bacterial cells were harvested by centrifugation (6000×*g*, 8 min, 4 °C). The harvested cells were resuspended in sterile water, adjusted to a final concentration of 1 × 10^10^ CFU/mL, and stored at 4 °C for subsequent use.

For the whole-target metabolomic analysis of strain BL21, fermentation broth was initially prepared at 4 °C, followed by vortexing for 1 min to ensure uniform mixing. An appropriate volume of the sample was then transferred to a 2 mL centrifuge tube and concentrated by drying. Subsequently, 500 μL of methanol solution was added to the dried sample, vortexed for 1 min, and centrifuged at 12,000 rpm for 10 min at 4 °C. The supernatant was carefully transferred to a new 2 mL centrifuge tube for a second drying concentration. Finally, the sample was reconstituted with 150 μL of a solution containing 80% methanol and 2-chloro-L-phenylalanine (4 ppm), and the resulting solution was filtered through a 0.22 μm filter into a vial for subsequent liquid chromatography-mass spectrometry (LC–MS) analysis^19^. Three independent samples of BL21 culture were analyzed by LC–MS to ensure the accuracy and reproducibility of the experimental results.

### Experimental animals

Zebrafish were maintained in water at 28 °C (water composition: 200 mg/L of instant sea salt of reverse osmosis water, conductivity of 450–550 μS/cm, pH of 6.5–8.5, and hardness of 50–100 mg/L CaCO_3_), provided by the Hunter Biotechnology Inc. (Hangzhou, China) aquaculture center. The use of experimental animals was licensed under permit number: SYXK (Zhe) 2022-0004, with husbandry management meeting the requirements of the international AAALAC accreditation (Accreditation number: 001458). Ethical review approval was granted by the Institutional Animal Care and Use Committee (IACUC) under the number: IACUC-2024-8511-3-01.

### Ethical statement

This study did not involve human participants or the use of human tissue samples. All experimental methods were strictly conducted in accordance with relevant ethical guidelines and regulations. Specifically, our experimental protocol was rigorously reviewed and approved by the Institutional Animal Care and Use Committee (IACUC) of Hunter Biotechnology Inc. The experiments were designed to minimize animal suffering and adhered to the institution’s animal welfare policies. Additionally, all methods are reported in compliance with the ARRIVE guidelines to ensure transparency and reproducibility in animal research. Further details on these guidelines are available at ARRIVE Guidelines (https://arriveguidelines.org).

### Experimental grouping and sample handling

This study used 6-month-old wild-type AB strain male zebrafish, which were randomly assigned into four groups (n = 10 per group): control (CTL), cyclophosphamide model (CS), BL21 treatment (BL21), and positive control (CC). To induce oligospermia^17^, all groups except CTL were exposed to cyclophosphamide (200 μg/mL) via waterborne exposure from day 1 to day 9. Following this period, the BL21 group received BL21 supplementation (3 × 10⁷ CFU/mL) from day 9 to day 16, while the CC group was treated with clomiphene citrate (0.100 μg/mL)^20^ for the same duration. All treatments, including cyclophosphamide, BL21, and clomiphene citrate, were administered via waterborne exposure, with daily solution replacement to maintain consistency^15^. Throughout the experiment, zebrafish were fed daily with a standard commercial diet, ensuring uniform nutrition across groups. The environmental conditions were maintained at 28 ± 0.5 °C with a 14-h light/10-h dark photoperiod. On day 16, zebrafish were euthanized using an overdose of tricaine methane sulfonate (MS-222, 200 mg/L), and testicular tissues were collected for further analyses.

### Effects of BL21 intervention on body and testis weights in zebrafish model

At the end of Day 16, the body weight of each zebrafish was measured using a precise electronic scale, and the testis weight was measured using a precision balance after dissection.

### Effects of BL21 intervention on sperm vitality and quality in zebrafish model

At the end of the intervention, zebrafish testes were collected and immediately transferred to 37 °C pre-warmed physiological saline solution, where they were incubated for 10 min to allow sperm release. The sperm suspension was then carefully collected and stored at low temperature in a preservation solution for subsequent analysis. The collected sperm samples were stained using a fluorescence staining kit specifically designed for zebrafish and observed under a Zeiss fluorescence microscope. Advanced image analysis was performed using ImageJ software to calculate the proportion and total number of viable sperm. Furthermore, sperm quality was assessed by measuring DNA integrity. On day 16 of the intervention, zebrafish testicular samples were collected and stained with acridine orange (AO). The DNA fragmentation of sperm was then analyzed using a flow cytometer, and the DNA Fragmentation Index (DFI) was recorded. This assessment of DNA integrity is a fundamental step in evaluating sperm functional capacity^21^ and provides deeper insights into the biological mechanisms underlying BL21’s impact on reproductive functions.

### Effects of BL21 intervention on the chasing behavior frequency of zebrafish towards female fish

On day 16, the chasing behavior of male zebrafish towards normal female zebrafish placed in the same tank was recorded for 30 min using a high-resolution camera. Before recording, male and female fish from each experimental group were placed in separate mating tanks at a 1:1 ratio, separated by a divider that allowed them to become acquainted through scent. Once the divider was removed, the chasing behavior commenced. During this process, male fish frequently chased the female from zone 1 to zone 2, where the female was forced to turn and swim back, slowing down and engaging in mating behavior with the male. The frequency of this chasing behavior reflects the male sexual function^22^.

### Effects of BL21 intervention on hormone levels in zebrafish model

At the end of experiment, testicular samples from zebrafish were collected. The concentrations of testosterone, luteinizing hormone (LH), and follicle-stimulating hormone (FSH) were determined using assay kits on a multifunctional enzyme reader.

### Effect of BL21 intervention on gene expression related to spermatogenesis and testicular function in zebrafish

Testis samples were collected from each group at the end of the treatment period. RNA was extracted from these samples using the Universal RNA Extraction TL Kit C. The quality and concentration of the extracted RNA were determined using a UV–Vis spectrophotometer. From the high-quality RNA, 2.00 μg was used to synthesize 20.0 μL of cDNA using the First Strand cDNA Synthesis Kit, following the manufacturer’s protocol. Quantitative PCR (q-PCR) was performed to assess the expression levels of genes essential for testicular function and spermatogenesis: β-actin (internal control), PCNA, NANOG, ZBTB16, mTOR, DDX4, CYP26A1, and ALDH1A2. The primer information involved in the experiment is shown in Table 1. The q-PCR conditions included an initial denaturation at 95 °C for 2 min, followed by 39 cycles of denaturation at 95 °C for 5 s and annealing/extension at 60 °C for 30 s each. Melting curve analysis: The temperature was raised from 60 to 95 °C, and the fluorescence values of the PCR products were recorded to plot the melting curve. The relative expression levels were calculated using the ΔΔCt method.

**Table 1:** Primer sequence information.

Gene	Primer type	Primer sequence	Accession no
β-actin	Forward	5′-TCGAGCAGGAGATGGGAACC-3′	NM_181601.5
	Reverse	5′-CTCGTGGATACCGCAAGATTC-3′	
PCNA	Forward	5′-TCATGATCTCGTGTGCCAAG-3′	NM_131404.2
	Reverse	5′-GGGCGCCAGGTAATATTTGA-3′	
Nanog	Forward	5′-CCGCAGGATGAAACTCAAGA-3′	NM_001098392.1
	Reverse	5′- GGTAACTGGGGTAGAAGGGT-3′	
ZBTB16	Forward	5′-CCTAAGGATGCACTTGCTGT-3′	NM_199635.1
	Reverse	5′-CTGATCCTGTGTGGATCTGC-3′	
mTOR	Forward	5′-AGCTGGAGTTTCAAAAGGGG-3′	NM_001077211.3
	Reverse	5′-TGCTTCCAGACATCTCATGC-3′	
DDX4	Forward	5′-CCTTCCTGCTGCCTATCCTA-3′	NM_131057.1
	Reverse	5′-CAACAGGTCGTACACAGGTC-3′	
CYP26A1	Forward	5′-AAGCTGGTGTCTGTTCAGTG-3′	NM_131146.2
	Reverse	5′-CATTCCTGTATGGCGCTCTT-3′	
ALDH1A2	Forward	5′-GTGTTGAGAGAGCACAGAGG-3′	NM_131850.1
	Reverse	5′-CTCCAGTTTGGCTCCTTCAG-3′	

### Statistical analysis

All results were expressed as mean ± standard deviation (Mean ± SD). Data analysis was performed using R (v 4.3.2), including one-way or multifactorial analysis of variance (ANOVA) to detect significant differences between treatment groups, with a p-value of less than 0.05 considered statistically significant.

## Results

### Metabolomic analysis of strain BL21

**Fig. 1:**
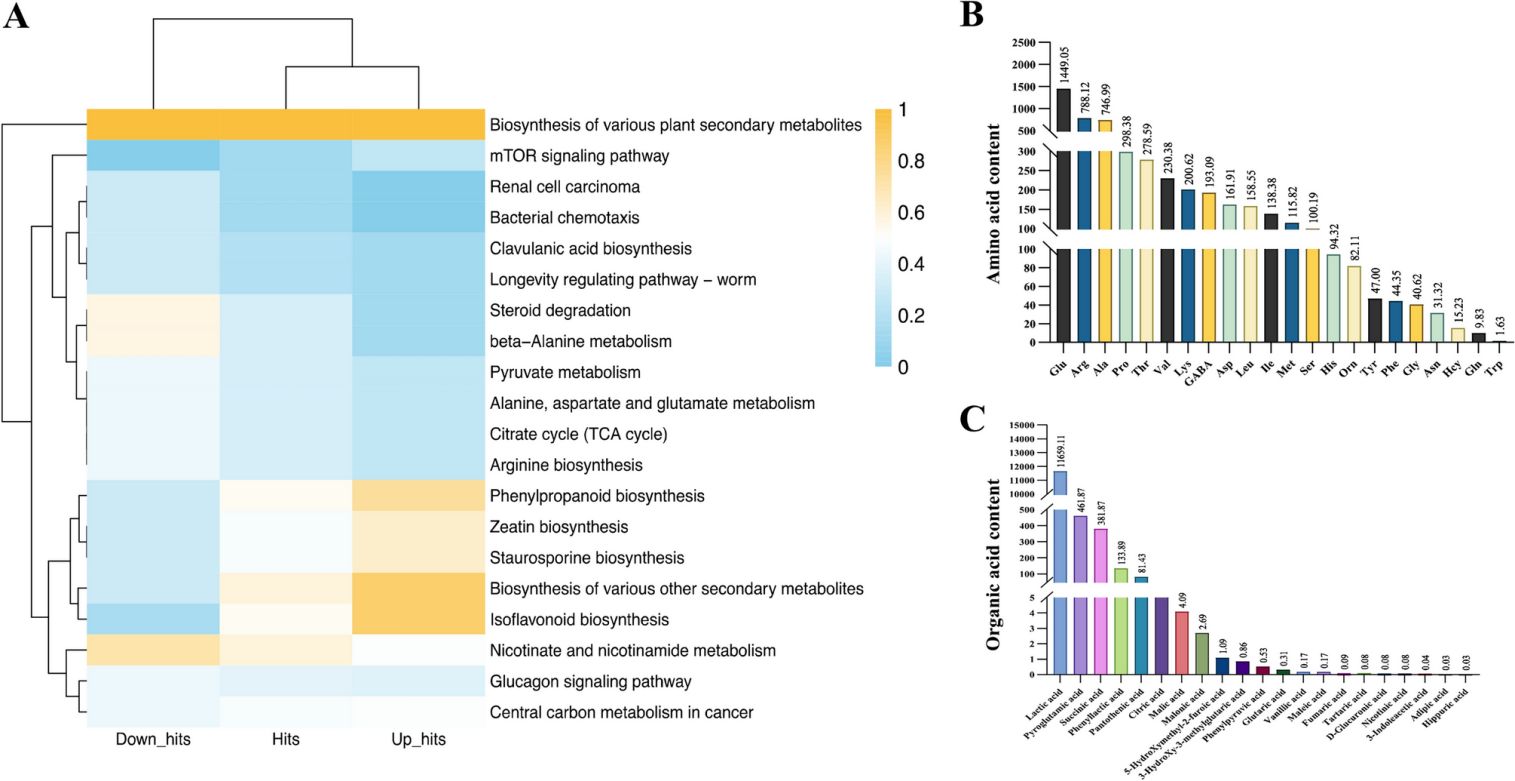
Metabolomic profiling of *Bifidobacterium longum* subsp. *longum* BL21. (**A**) Pathway analysis displays pathways significantly influenced by the fermentation product. “Up_hits” are pathways with increased activity (blue), “Down_hits” denote pathways with decreased activity (yellow), and “Hits” are pathways with detected activity that has not significantly changed. (**B**) Amino acid and (**C**) organic acid levels show the concentrations of various amino acids and organic acids contained in the inactivated bacterial mass.

In our targeted metabolomic analysis of the BL21 strain fermentation broth, significant modulation was observed in multiple key metabolic pathways (Fig. 1A). Specifically, pathways such as the biosynthesis of secondary metabolites, antioxidant and energy metabolism-related pathways, demonstrated substantial upregulation, while pathways including pyruvate metabolism, bacterial chemotaxis, and amino acid metabolic processes exhibited significant downregulation. In the targeted amino acid analysis following the pasteurization of the BL21 strain (Fig. 1B,C), we detected a total of 22 amino acids. Among these, glutamate (1449.05 μg/g), arginine (788.12 μg/g), and glycine (425.19 μg/g) were present at relatively high concentrations. In addition, 19 organic acids were identified, with lactate (11,659.11 μg/g), pyrrolidone carboxylic acid (461.87 μg/g), and succinic acid (381.87 μg/g) being the most abundant. The presence of these metabolites, particularly amino acids like glutamate and arginine and organic acids such as lactate and succinic acid, highlights their potential roles in energy metabolism regulation, antioxidant activity, and cellular function enhancement. Collectively, these results indicate that BL21 metabolites may have beneficial effects on metabolic homeostasis and oxidative stress alleviation, thus supporting improved reproductive health outcomes.

### Effects of BL21 intervention on reproductive disorders in zebrafish: body and testicular weight

**Fig. 2:**
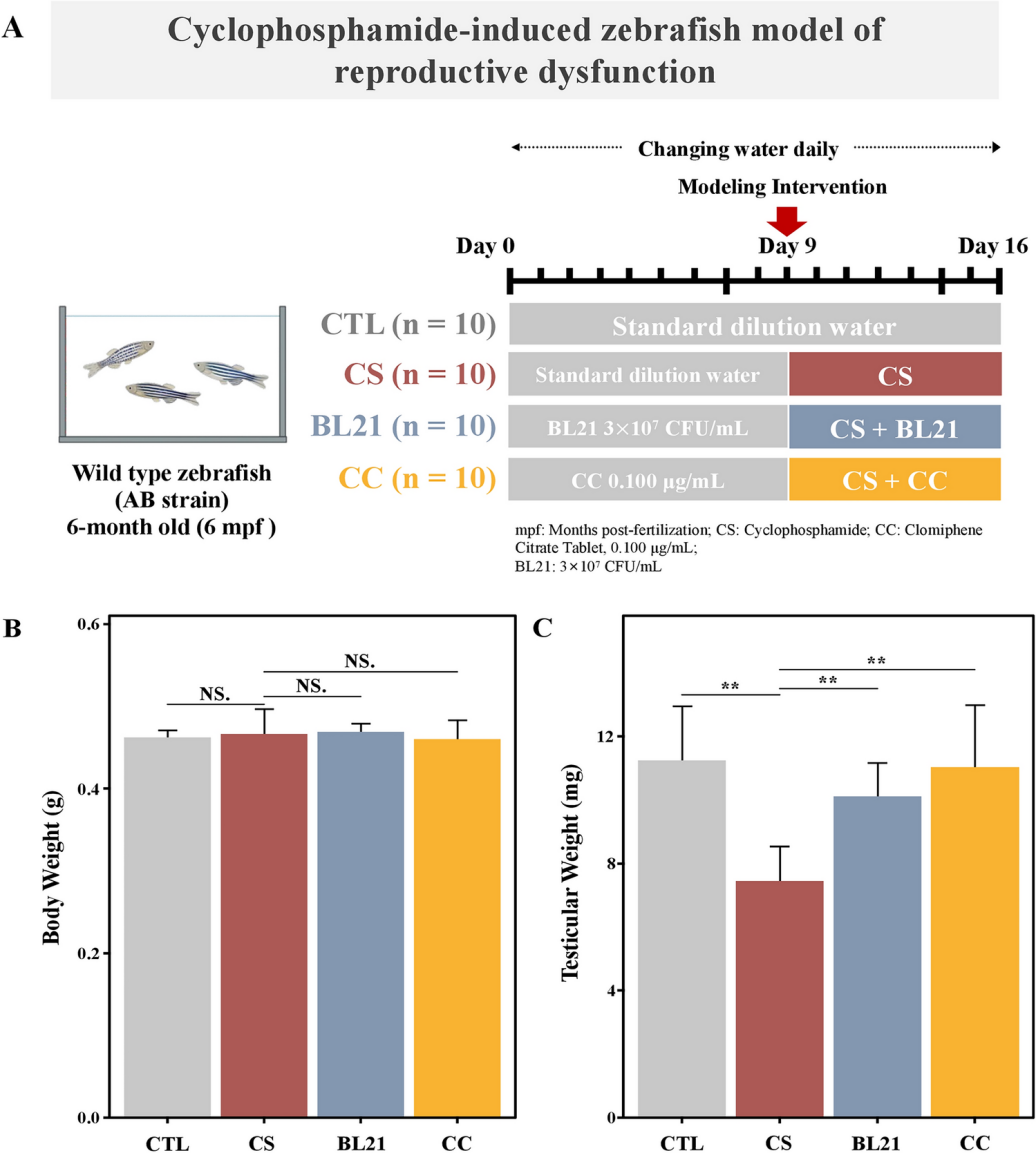
Experimental design to evaluate the impact of *Bifidobacterium longum* subsp. *longum* BL21 on cyclophosphamide-induced reproductive dysfunction in zebrafish. (**A**) Experimental flowchart detailing the procedural steps, (**B**) Body weight comparisons, (**C**) Testicular weight measurements across different treatment groups: CTL (normal control), CS (cyclophosphamide-only), BL21 (BL21 treatment), and CC (clomiphene citrate as positive control). NS indicates no significant difference. ** *p* < 0.01.

The experimental procedure is shown in Fig. 2A. Results indicated that there was no significant change in the body weight of zebrafish across all experimental groups (Fig. 2B), suggesting that BL21 intervention does not affect the overall physique of the zebrafish. However, when comparing testicular weight (Fig. 2C), zebrafish treated with BL21 and the positive control drug CC showed a significant increase in testicular weight (both *p* < 0.01) compared to CS group, with results statistically similar to CTL group. This indicates that BL21 supplementation helps mitigate reproductive dysfunction induced by the model conditions, with statistical significance comparable to the effects of the positive control drug, highlighting the efficacy of BL21 as a potential reproductive health enhancer.

### Effects of BL21 intervention on sperm viability in zebrafish with reproductive disorders

**Fig. 3:**
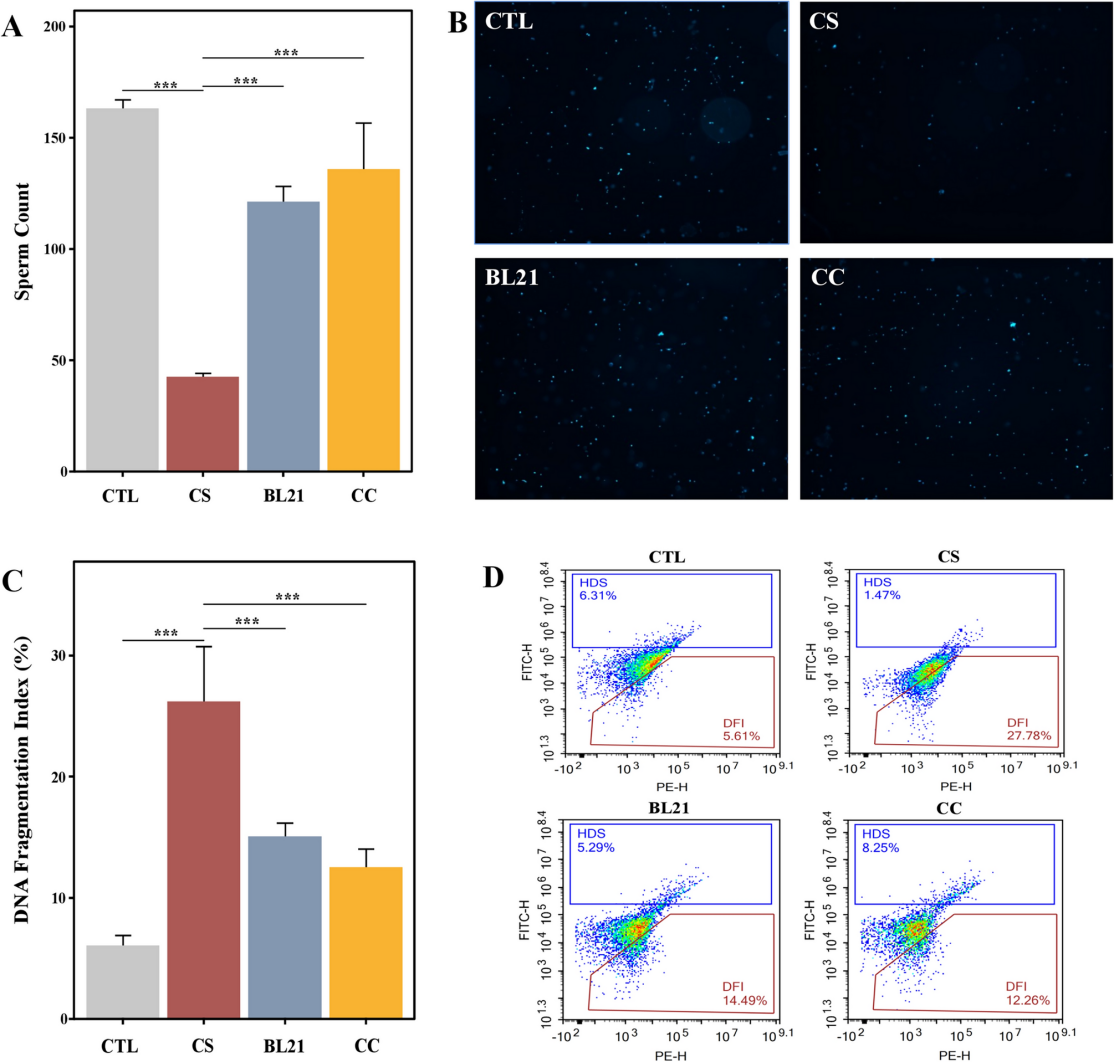
Impact of *Bifidobacterium longum* subsp. *longum* BL21 on sperm parameters in zebrafish experiencing cyclophosphamide-induced reproductive dysfunction. (**A**) Sperm count, (**B**) Viability, (**C**,**D**) DNA fragmentation. The groups are as follows: CTL (normal control), CS (cyclophosphamide-only), BL21 (BL21 treatment), and CC (positive control with clomiphene citrate). ***p < 0.001. FITC-H denotes fluorescence intensity of stained DNA, reflecting DNA fragmentation levels, and SSC-H represents side scatter height, assessing cellular complexity.

Figure 3A illustrates that in CS group, the total sperm count was significantly reduced compared to CTL group (*p* < 0.001). However, in groups treated with BL21 and CC, the sperm count was significantly higher than in the CS group (*p* < 0.001), demonstrating BL21’s effectiveness in promoting sperm production in damaged sperms. Additionally, fluorescence staining results (Fig. 3B) revealed that sperm viability was significantly decreased in CS group, as indicated by reduced fluorescence intensity. In contrast, sperm viability was significantly enhanced in zebrafish treated with BL21 and CC, with a noticeable increase in fluorescence intensity. These quantitative and qualitative observations collectively confirm that BL21 is not only effective in increasing sperm count but also in enhancing the biological activity and function of sperms, showcasing its potential application advantages.

### Impact of BL21 intervention on DNA fragmentation in zebrafish sperm with reproductive disorders

Flow cytometry was employed to quantitatively analyze DNA fragmentation in zebrafish sperm (Fig. 3C,D), assessing the impact of BL21 intervention on sperm DNA integrity. Figure 3C illustrates that, compared to CTL, CS group exhibited significantly increased sperm DNA fragmentation, indicating that the model successfully induced reproductive dysfunction. However, after treatment with the probiotic BL21 and the positive control drug CC, the level of sperm DNA fragmentation significantly decreased (*p* < 0.001), approaching the levels seen in CTL group. This suggests that these interventions effectively restored the integrity of sperm DNA. Flow cytometric analysis (Fig. 3D) showed that in the CTL group, most cells were located in the DFI area (5.61%), indicating lower DNA fragmentation. In contrast, in the CS group, a large number of cells accumulated in the DFI area (27.78%), reflecting a high state of DNA fragmentation. Treatment with BL21 and CC significantly increased the proportion of cells in the DFI area to 14.49% and 12.26% respectively, demonstrating that these interventions can significantly reduce DNA damage and restore the health of sperm.

### Regulation of male zebrafish chasing behavior by BL21 intervention

**Fig. 4:**
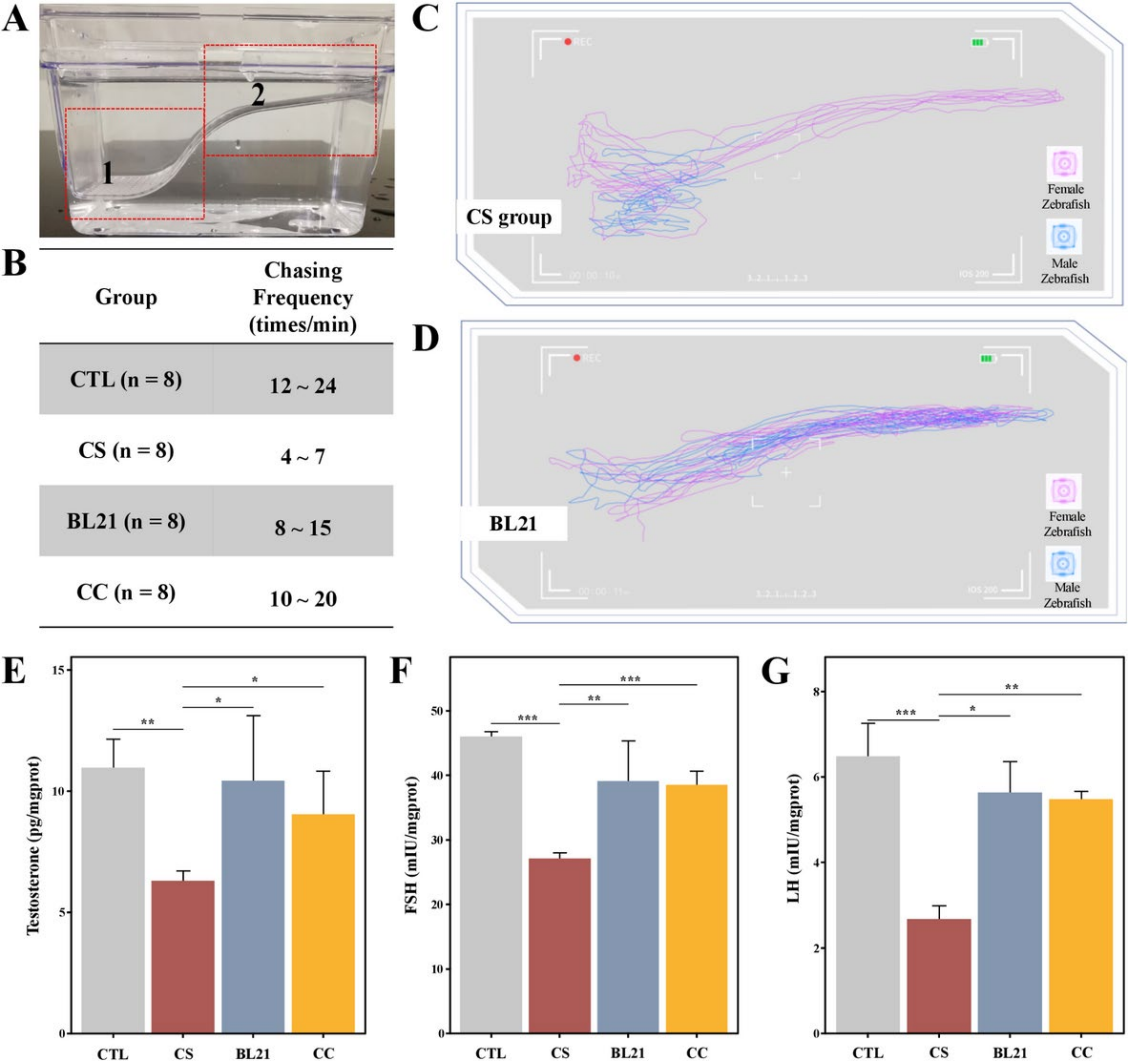
Effects of *Bifidobacterium longum* subsp. *longum* BL21 on reproductive behaviors and hormonal responses in zebrafish. (**A**) Diagram of the experimental tank setup, divided into a starting zone ('1') and a chasing zone ('2') to assess zebrafish mating behaviors. (**B**) Table presenting the frequency of male zebrafish chasing behaviors per minute across four groups: Control (CTL), Cyclophosphamide-only (CS), treated with BL21 (BL21), and a Clomiphene Citrate positive control (CC). (**C**) Movement trajectory plots for male (blue) and female (pink) zebrafish in the CS group showing baseline activities. (**D**) Enhanced trajectories in the BL21 group illustrate more frequent and interactive chasing, indicating improved reproductive behaviors after treatment. (**E**) Testosterone levels graph comparing responses across all groups, highlighting hormonal adjustments following treatments. (**F**) Follicle-stimulating hormone (FSH) levels graph, demonstrating gonadotropic responses to the treatments. (**G**) Luteinizing hormone (LH) levels graph, detailing endocrine responses across the treatment groups. * *p* < 0.05, ** p < 0.01, *** p < 0.001.

Male zebrafish were paired with normal female zebrafish at a 1:1 ratio in mating tanks equipped with dividers. This setup allowed the fish to familiarize themselves with each other’s scent through the divider, which was removed to start the observation of the male’s chasing behavior (Fig. 4A). This behavior involved males chasing females from area 1 to area 2, where the females would turn and swim back, slowing down, and mating behavior would occur. The frequency of male zebrafish chasing is an important indicator of male sexual behavior activity. Results (Fig. 4B) showed that in CTL group, the chasing frequency ranged from 12 to 24 times/min. In contrast, in CS group, the chasing frequency significantly decreased to 4 to 7 times/min, indicating that the model successfully induced a decline in reproductive behavior. However, after intervention with BL21, the chasing frequency improved to 8 to 15 times/min, marking an improvement in behavior, although still lower than CTL group, but comparable to the positive control drug group CC and higher than CS group. The intervention with BL21 could partially restore the sexual behavior activity impaired by reproductive disorders.

Combining Fig. 4C,D, we observed behavioral differences between the two different treatment groups (CS and BL21). The tracking paths clearly displayed the movement patterns of male and female zebrafish, with blue lines representing males and red lines representing females. In the CS group (Fig. 4C), the movement trajectories of the males (blue lines) showed less activity and maintained a greater distance from the females’ trajectories (red lines). This indicated a reduction in chasing behavior in the CS group, consistent with an impaired reproductive behavior phenotype. The males’ paths were dispersed with no evident chasing or proximity to the females, likely reflecting a decrease in sexual behavior activity. In contrast, in Fig. 4D, the movement trajectories of the males (blue lines) more frequently intersected with the females’ trajectories (red lines), displaying clear chasing behavior. The males followed the females closely, exhibiting persistent chasing actions, a typical sign of active sexual behavior. This behavioral pattern suggests that BL21 intervention may effectively restore male reproductive behavior activity, enhancing their attraction and chasing ability towards females.

### Improvement of reproductive hormone levels in zebrafish with reproductive disorders by BL21 intervention

We explored the effects of BL21 intervention on the hormone levels of zebrafish with impaired reproductive functions (Fig. 4E–G). The results showed that in CS group, the levels of testosterone, LH, and FSH significantly decreased (*p* < 0.01, *p* < 0.001 and *p* < 0.001, respectively), confirming the effectiveness of the experimental model in inducing reproductive dysfunction. However, after treatment with BL21, these hormone levels significantly increased (*p* < 0.05, *p* < 0.01, and *p* < 0.05, respectively), slightly surpassing the effects of the traditional positive control drug CC. Specifically, the testosterone, LH, and FSH levels in the BL21 treatment group were significantly higher than those in CS group and performed better than the positive control drug group. These findings underscore the potential application of BL21 in regulating sex hormones and restoring reproductive functions.

### Impact of BL21 on gene expression related to spermatogenesis and testicular development in zebrafish

**Fig. 5:**
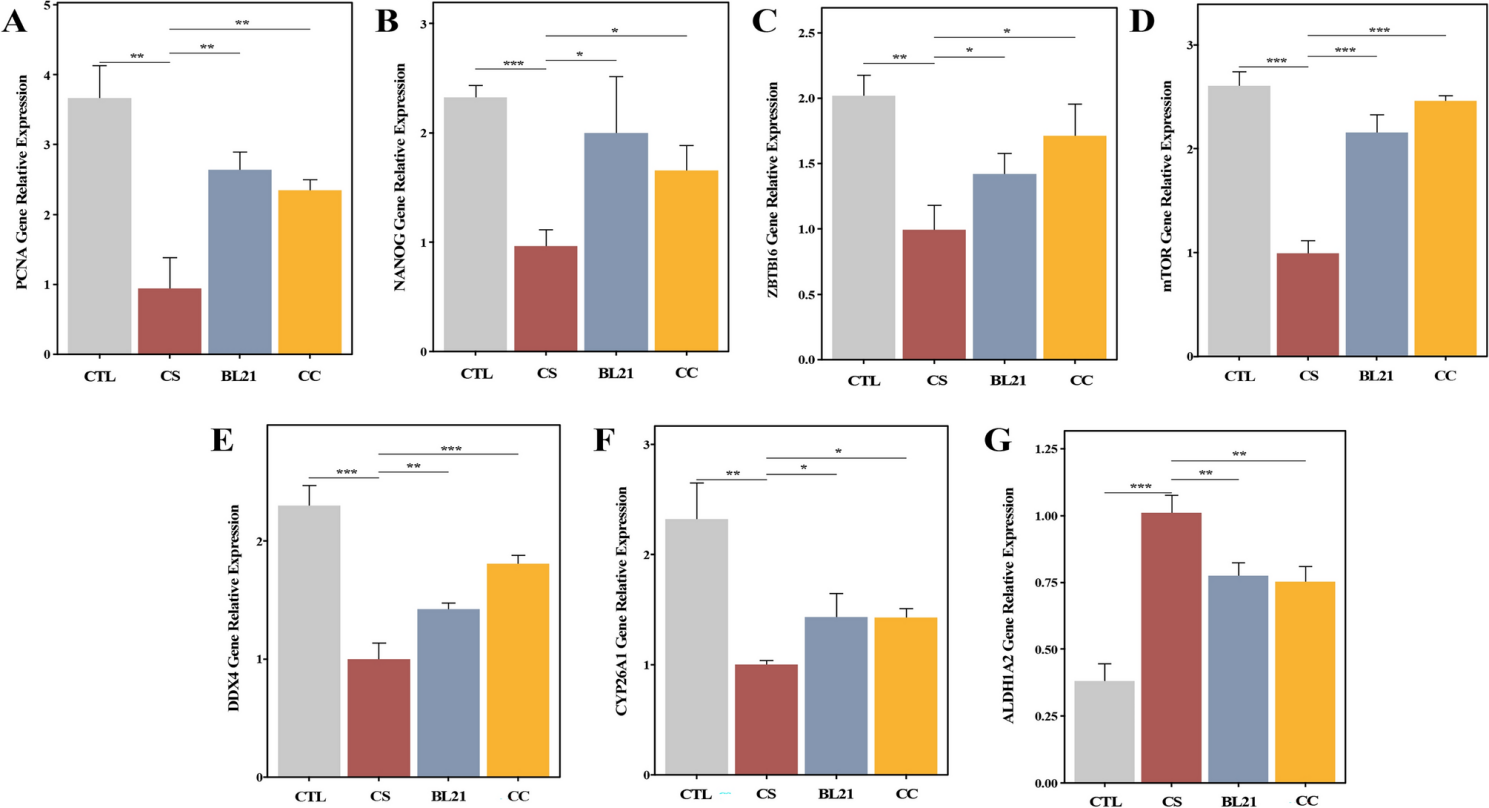
Impact of *Bifidobacterium longum* subsp. *longum* BL21 on gene expression related to spermatogenesis and testicular development in zebrafish. (**A**) PCNA, (**B**) NANOG, (**C**) ZBTB16, (**D**) mTOR, (**E**) DDX4, (**F**) CYP26A1, (**G**) ALDH1A2 gene expression. Groups are control (CTL), cyclophosphamide only (CS), BL21 treated (BL21), and clomiphene citrate positive control (CC). *p < 0.05; **p < 0.01; ***p < 0.001.

As depicted in Fig. 5A–G, compared to the control (CTL) group, the cyclophosphamide-induced model control group (CS) exhibited significant reductions in the expression levels of PCNA, NANOG, ZBTB16, mTOR, DDX4, and CYP26A1 (all *p* < 0.01), while ALDH1A2 expression was significantly elevated (*p* < 0.001), indicating successful model induction. Conversely, in groups treated with BL21 and the positive control drug, there was a reversal in gene expression levels: significant increases were observed in the expression of PCNA, NANOG, ZBTB16, mTOR, and DDX4 (all *p* < 0.05), and a significant decrease in ALDH1A2 expression (*p* < 0.01). Notably, the regulatory effects of BL21 on the levels of PCNA and NANOG were more pronounced than those observed with the positive control drug. These findings suggest that intervention with BL21 can significantly modulate the expression of these key genes, potentially aiding in the restoration of spermatogenesis and testicular function.

## Discussion

**Fig. 6:**
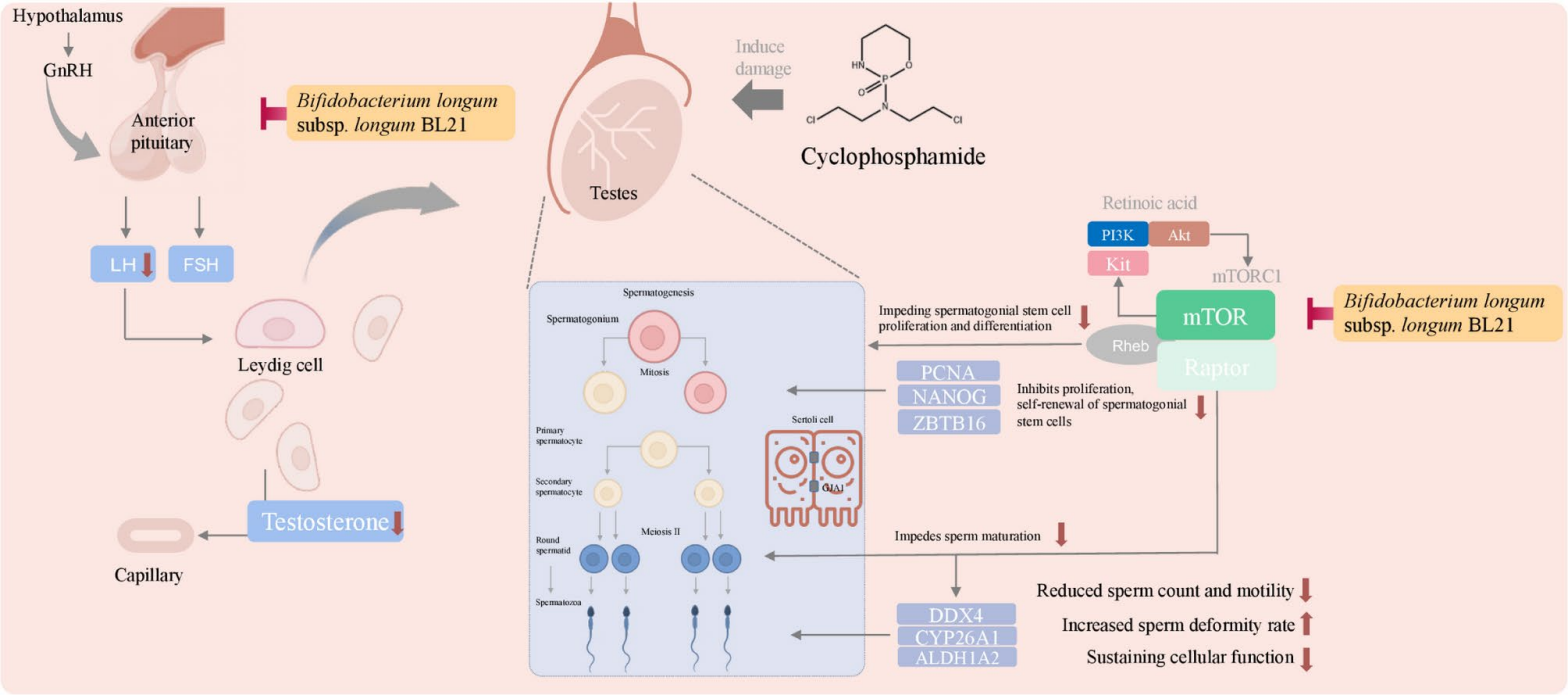
Effects of *Bifidobacterium longum* subsp. *longum* BL21 on reproductive recovery in cyclophosphamide-treated zebrafish.

In this study, we aimed to explore the potential effects of BL21 on reproductive dysfunction in zebrafish (Fig. 6). In recent years, probiotics have gained increasing attention for their potential in promoting host health, particularly in enhancing reproductive health^23^. Compared to traditional treatments that may induce side effects such as cardiovascular risks, infections, pain, hot flashes, and osteoporosis, probiotics offer a low-risk alternative^12^. Due to their reproductive system similarities and high manipulability, zebrafish are widely used in reproductive biology and related disease research. Initially, our study explored the potential metabolic functions of BL21 through in vitro biochemical pathway analysis applied to a zebrafish model of reproductive disorders. Subsequently, we investigated its effects on zebrafish testis weight and sperm vitality, as well as the modulation of sex hormone levels, revealing BL21’s positive impact on multiple biological levels of zebrafish reproductive functions. The experimental results not only confirmed BL21’s effectiveness in enhancing damaged sperm production and improving sperm vitality but also demonstrated its role in regulating sex hormone levels and reducing sperm DNA fragmentation. Interestingly, the intervention effects of the probiotic BL21 were comparable to conventional medications, and early studies indicated it has no significant side effects^24^. Therefore, these findings provide a crucial foundation for further research into how probiotics like BL21 could improve reproductive health through biochemical mechanisms.

In zebrafish model, we explored the effects of BL21 on testicular weight and sperm count, finding that BL21 significantly enhanced these key reproductive health indicators. The increase in testicular weight may reflect improved proliferation and health of gonadal cells, while the increase in sperm count directly indicates enhanced reproductive capacity^25^. These effects are associated with non-target metabolites in the BL21 fermentation broth, such as the activation of the mTOR signaling pathway. The mTOR signaling pathway is a crucial regulator of cellular growth and metabolism, and its activation is closely linked to cell proliferation, survival, and biosynthesis processes^26^. Thus, the activation of this pathway by BL21 may promote the proliferation and survival of zebrafish testicular cells, providing support for the recovery of damaged sperm. Further, BL21 intervention significantly increased zebrafish sperm vitality and improved the condition of sperm DNA fragmentation. Sperm from zebrafish treated with BL21 exhibited higher fluorescence intensity, reflecting a lower rate of DNA fragmentation and healthier sperm condition^21,27^. Combined with the results of the whole-target metabolomic analysis of BL21, upregulation of the mTOR pathway could have a positive impact on sperm biosynthesis and cellular survival mechanisms. The activation of the mTOR pathway, by enhancing the metabolic activity and energy production of sperm cells, aids in improving the antioxidant defense capacity of sperm, thereby reducing DNA damage caused by oxidative stress^28^. Additionally, since the mTOR pathway plays a central role in cellular proliferation and growth^26^, its activation may promote the quantity and quality of sperm, providing a robust repair mechanism to maintain DNA integrity. Furthermore, the enhancement of isoflavone biosynthesis could also provide another layer of protection for sperm. Isoflavones, potent natural antioxidants^29^, combat oxidative damage caused by free radicals, which is crucial for preventing sperm DNA fragmentation. Through this mechanism, BL21 could indirectly protect sperm from harmful metabolic by-products by increasing intracellular antioxidant levels. Meanwhile, the enhanced biosynthesis of plant secondary metabolites suggests that BL21 may improve sperm health through multiple mechanisms. These metabolites typically include polyphenols and flavonoids^30^, which can directly neutralize harmful oxidants and may also improve the survival environment for sperm by regulating local hormone levels and cellular responses^31^.

In behavioral experiments, male zebrafish treated with BL21 exhibited increased pursuit behavior toward female zebrafish following barrier removal. This enhancement in behavior is not only a direct indicator of increased reproductive capacity but also reflects an overall increase in sexual activity^22^. These behavioral changes may, to some extent, be associated with specific metabolites produced by BL21 that influence neurotransmitter release or improve the endocrine environment, thereby enhancing the motivation and capability for sexual behavior. Neurotransmitters such as dopamine and serotonin are known to impact reproductive behaviors and the functioning of reproductive organs, particularly their roles in regulating the release of sex hormones and the maturation of sperm^32,33^. The observed increase in sexual activity could be linked to the components in the BL21 fermentation broth improving the endocrine environment, such as by increasing testosterone levels, which in turn enhances the sexual drive and mating behavior of male fish. Monitoring critical hormone levels, specifically testosterone, LH, and FSH, is essential as these hormones play central roles in reproductive function regulation^34,35^. The effects of BL21 on these hormone levels directly reflect its potential to improve reproductive health. Testosterone, a key hormone dominant in male reproductive health, is responsible for maintaining libido, reproductive organ development, and normal spermatogenesis^36^. Results indicate that BL21 significantly raises testosterone levels in a zebrafish model of reproductive dysfunction, suggesting that BL21 may enhance testosterone production either by supporting the inherent hormone synthesis mechanisms of the testes or by modulating downstream signaling pathways. Moreover, as gonadotropins, LH and FSH directly regulate the synthesis of testosterone and sperm production^37^. The significant elevation in LH and FSH levels associated with BL21 treatment may reflect the probiotic’s positive modulation of the gonadotropic axis. Compared to traditional positive control drugs like clomiphene citrate, frequently used to stimulate the secretion of LH and FSH thereby elevating testosterone levels^38^, BL21 shows similar efficacy in hormone regulation. However, BL21’s unique advantage likely lies in its ability to regulate these hormones through more natural mechanisms rather than simple pharmacological stimulation, potentially leading to more balanced hormone level changes and lower side effect risks. For example, BL21 may enhance hormonal balance naturally by improving the gut microbiota environment and/or directly affecting the gut-brain-gonadal axis. This regulatory mechanism not only reveals how BL21 improves impaired reproductive health but also highlights how it maintains or restores natural hormonal homeostasis without causing excessive physiological stress responses. Linked to the metabolomic analysis results of the BL21 fermentation broth, the activation of the isoflavone biosynthesis pathway plays a role in hormone balance and antioxidant defense^39^. Thus, we speculate that BL21 may enhance this pathway’s activity, potentially mimicking estrogen effects to improve male zebrafish reproductive health. This discussion sheds light on the multifaceted mechanisms through which BL21 might improve reproductive health and underscores its potential as a safe and effective alternative to conventional treatments.

In our study, we investigated how the probiotic BL21 regulates genes associated with spermatogenesis such as PCNA, NANOG, ZBTB16, mTOR, DDX4, CYP26A1, and ALDH1A2, and their impact on biological processes. PCNA and NANOG are crucial for cell proliferation and stem cell renewal, playing key roles in maintaining healthy spermatogenesis and testicular function^40^. BL21 treatment significantly enhanced the expression of these genes, suggesting that the probiotic activates important regulators of cell proliferation and survival, thereby enhancing the proliferation of spermatogonia and maturation of sperm, and aiding in the restoration of impaired reproductive functions. Notably, BL21 activated the mTOR signaling pathway, a key regulator of cell growth and metabolism, potentially improving protein synthesis and metabolic activities^41^, thus enhancing the proliferative capacity and vitality of reproductive cells. This finding aligns with our non-targeted metabolomic analysis, which showed a significant upregulation of the mTOR pathway. Additionally, the detection of relatively high concentrations of amino acids like glutamate and arginine, which are crucial for protein synthesis and various metabolic pathways, suggests a positive effect on sperm energy metabolism. Glutamate serves as a precursor to neurotransmitters and is involved in key energy production pathways, while arginine, a semi-essential amino acid, is critical for the production of nitric oxide (NO), which plays a role in regulating vasodilation and improving blood circulation to reproductive organs, potentially affecting sperm quality and vitality. Moreover, the increased expression of ZBTB16 and DDX4 is crucial for the accurate positioning and differentiation of sperm precursor cells^42^, with BL21 likely regulating these key RNA-binding proteins to foster sperm maturation and function. Our findings indicate that BL21 treatment significantly impacts the expression of the retinoic acid-metabolizing enzymes CYP26A1 and ALDH1A2, helping to maintain the levels of retinoic acid necessary for spermatogenesis^43^. The influence of BL21 on these enzymes can indirectly modulate the activity and bioavailability of sex hormones, which are vital for spermatogenesis, gonadal function, and overall reproductive health. By adjusting retinoic acid levels, BL21 may influence hormone receptor expression, enhancing sperm maturation and increasing reproductive success rates.

Furthermore, although our metabolomic results from BL21 indicate a downregulation of some key pathways in the TCA cycle, such as pyruvate metabolism and beta-alanine metabolism, we also observed increased concentrations of specific intermediates like pyrrolidone carboxylic acid and succinic acid. This suggests that, despite partial suppression of the overall TCA cycle, cells may compensate by adjusting other metabolic pathways to maintain necessary energy output, meeting the energy needs of sperm and their precursor cells. For instance, lactate, a key end product of cellular metabolism, when present in high concentrations locally, may indicate active cell metabolism, which is beneficial for sperm energy supply^44–46^. Additionally, moderate concentrations of pantothenic acid (vitamin B5) and citric acid also play roles in maintaining sperm metabolism and adapting to external environmental stress^47^, further illustrating the metabolic adaptability and flexibility exhibited by cells under the influence of BL21. By adjusting specific metabolic segments, BL21 may help sperm adapt to various biological needs. These insights emphasize BL21’s capability to enhance sperm quantity and quality through multi-pathway synergistic actions, providing a solid scientific foundation for probiotic-based reproductive health therapies. Not only does this confirm BL21’s potential to promote spermatogenesis and improve male reproductive health, but it also highlights the complex role probiotics play in regulating intricate reproductive biological processes.

To summarize, this study highlights the potential of BL21 in enhancing reproductive functions in zebrafish, offering strong preliminary evidence for probiotics as reproductive health interventions. We detailed how BL21 influences hormone regulation and sperm vitality, uncovering potential mechanisms. However, limitations include the lack of long-term safety and efficacy data, absence of clinical validation in human studies, and the absence of histopathological analysis, which restricts direct insights into tissue-level effects. Future research should include comprehensive histopathological evaluations and clinical trials to fully validate and extend these promising findings.

## Conclusion

In this study, we investigated the effects of BL21 on reproductive dysfunction in zebrafish. Results demonstrated that BL21 significantly enhanced hormone levels, testicular weight, sperm vitality, and reduced sperm DNA fragmentation, indicating its potential benefits in promoting reproductive health. Overall, this research provides valuable insights into the application of probiotics in reproductive health and paves the way for future therapeutic strategies.

## Data Availability

All data generated or analyzed during this study are included in this article.

## Abbreviations

CS: Cyclophosphamide
CC: Clomiphene citrate
LH: Luteinizing hormone
FSH: Follicle-stimulating hormone
PCNA: Proliferating cell nuclear antigen
NANOG: NANOG homeobox
ZBTB16: Zinc finger and BTB domain containing 16
mTOR: Mammalian target of rapamycin
DDX4: DEAD-box helicase 4
CYP26A1: Cytochrome P450 family 26 subfamily A member 1
ALDH1A2: Aldehyde dehydrogenase 1 family member A2

## Declarations

### Compliance with ARRIVE guidelines

This study was carried out in strict accordance with the recommendations in the ARRIVE guidelines for reporting experiments involving animals. The full ARRIVE guidelines checklist has been reviewed, and the methods and results are presented in accordance with these guidelines.
